# Iron (II/III) perchlorate electrolytes for electrochemically harvesting low-grade thermal energy

**DOI:** 10.1038/s41598-019-45127-w

**Published:** 2019-06-18

**Authors:** Ju Hyeon Kim, Ju Hwan Lee, Ramasubba Reddy Palem, Min-Soo Suh, Hong H. Lee, Tae June Kang

**Affiliations:** 1 Department of Mechanical Engineering, INHA University, Incheon, 22212 South Korea; 2 Energy Efficiency and Materials Research Division, Korea Institute of Energy Research, Daejeon, 34129 South Korea; 3 School of Chemical and Biological Engineering, Seoul National University, Seoul, 151-744 South Korea

**Keywords:** Devices for energy harvesting, Electrochemistry

## Abstract

Remarkable advances have recently been made in the thermocell array with series or parallel interconnection, however, the output power from the thermocell array is mainly limited by the electrolyte performance of an n-type element. In this work, we investigate iron (II/III) perchlorate electrolytes as a new n-type electrolyte and compared with the ferric/ferrous cyanide electrolyte at its introduction with platinum as the electrodes, which has been the benchmark for thermocells. In comparison, the perchlorate electrolyte (Fe^2+^/Fe^3+^) exhibits a high temperature coefficient of redox potential of +1.76 mV/K, which is complementary to the cyanide electrolyte (Fe(CN)_6_^3−^/Fe(CN)_6_^4−^) with the temperature coefficient of −1.42 mV/K. The power factor and figure of merit for the electrolyte are higher by 28% and 40%, respectively, than those for the cyanide electrolyte. In terms of device performance, the thermocell using the perchlorate electrolyte provides a power density of 687 mW/m^2^ that is 45% higher compared to the same device but with the cyanide electrolyte for a small temperature difference of 20 °C. The advent of this high performance n-type electrolyte could open up new ways to achieve substantial advances in p-n thermocells as in p-n thermoelectrics, which has steered the way to the possibility of practical use of thermoelectrics.

## Introduction

Low-grade heats generated from geothermal reservoirs, power plants and various industrial processes are recycled at low efficiency or just released to the surrounding environment in the form of waste heat^1–3^. Considering that almost two thirds of all the energy produced is discharged as waste heat, technologies are critically needed that can effectively convert this low-grade heat to useful electrical energy. Thermoelectric (TE) devices have extensively been studied in the past decades^4–6^ to convert the waste heat to electric energy. The TE devices do provide an effective means of converting waste heat to electricity. Rarity of the TE raw materials and the corresponding high unit cost of electric power, however, hampered its practical and large-scale deployment^7,8^. The small Seebeck coefficient typical of these TE materials (several tens to hundreds of μV/K)^9–11^ limits the generation of practically usable voltage from the TE devices because of the small temperature difference typically prevailing between waste heat source and its surrounding.

Other routes to utilizing waste thermal energy include thermoelectrochemical cells (TECs)^12–18^, thermally regenerative electrochemical cells (TRECs)^19–21^ and thermo-osmotic energy conversion (TOEC)^22,23^. Among these, TECs or thermocells have received much attention due to the advantages of direct conversion of thermal to electrical energy and continuous direct current (DC) voltage generation without consuming materials or producing emissions. In particular, the high temperature coefficient of redox potential on the order of mV/K makes TEC technologies attractive for utilizing waste thermal energy.

A TEC is a non-isothermal electrochemical cell with a simple structure of an electrolyte sandwiched between two non-active electrodes subjected to different temperatures. Studies on TECs have mainly centered around the electrolyte of ferric/ferrous cyanide (Fe(CN)_6_^3−^/Fe(CN)_6_^4−^) on the strength of a high temperature coefficient of −1.42 mV/K since its introduction in 1976^24^ with platinum electrodes, which has been the benchmark electrolyte since then. A temperature coefficient of redox potential (α) is related to the voltage that can be obtained from the cell at a given temperature difference and at constant pressure, which can be expressed as eq. (1)

1 $$\alpha ={\frac{\Delta V}{\Delta T}|}_{p}=\frac{\Delta {s}_{rx}^{0}}{nF}$$

where ΔV is the full-cell voltage, ΔT is the inter-electrode temperature difference, Δs°_rx_ is the entropy change for the redox reaction, n is the number of electrons transferred in the reaction, and F is Faraday’s constant. With the efforts on developing inexpensive but highly efficient electrodes and high-performance electrolytes, remarkable advances have been made in the TEC performance, which render TEC technologies commercially attractive^25^ (see Supplementary involving recent advances in thermocell performance).

In addition to these efforts to address the material requirements of a TEC device, studies on TEC arrays with series and/or parallel interconnections have been conducted to provide the required voltage and current for practical purposes. As being analogous to a commercial thermoelectric module utilizing p-type and n-type thermoelectric materials, an array of p-type and n-type thermocell elements has been demonstrated using electrolytes with opposite temperature coefficients. It should be noted that, considering the direction of temperature gradient and electric potential difference between hot and cold electrodes, taking the negative sign of α determines the type of TEC element. This provides a sign convention which is consistent with thermoelectrics for n-type and p-type elements in TECs, providing negative and positive temperature coefficients, respectively.

Combined TECs using different electrolytes of potassium ferric/ferrous cyanide (−α of +0.95 mV/K, termed a p-type electrolyte) and ferric/ferrous sulfate with 0.1 M H_2_SO_4_ (−α of −0.54 mV/K, termed an n-type electrolyte) was recently investigated to boost output voltage without introducing a thermal short-circuit^26^. Using gel electrolytes of poly(vinyl alcohol) with ferric/ferrous cyanide (+1.21 mV/K) and ferric/ferrous chloride (−1.02 mV/K) redox couples, a flexible and wearable TEC array was fabricated for the purpose to utilize body heat^27^. A TEC array containing potassium ferric/ammonium ferrous cyanide (+1.43 mV/K) and ferric/ferrous sulfate (−0.5 mV/K) electrolytes was also demonstrated to charge commercial capacitors utilizing low-grade thermal energy^18^.

Although exciting recent progress has been realized for the TEC array with series and parallel interconnection, the output performance from the TEC array was found to be mainly limited by a relatively low performance of n-type electrolytes, compared to that of p-type electrolytes, at the use of same cross-sectional areas and electrode materials of both n-type and p-type elements. Therefore, a new electrolyte that compliments and/or outperforms the cyanide electrolyte could open up new ways to achieve substantial advances in p-n TECs as in p-n thermoelectrics, which has steered the way to the possibility of practical use of thermoelectrics.

In this work, we introduce an aqueous electrolyte of iron (II/III) perchlorate (Fe(ClO_4_)_2_/Fe(ClO_4_)_3_) as a high-performance, n-type electrolyte for TECs that harvest thermal energy at temperatures below 100 °C. For direct comparisons between electrolytes, we adopt the typical measures of performance of thermoelectrics for the electrolytes such as power factor and figure of merit. The intrinsic quantities pertaining to these measures are temperature coefficient of redox potential, ionic conductivity, and thermal conductivity. To deliver high electrical power, it is desirable for an electrolyte to have the high temperature coefficient, high ionic conductivity, and low thermal conductivity. We determine experimentally these quantities and compare the proposed perchlorate electrolyte against the benchmark cyanide electrolyte in terms of the ionic power factor and figure of merit. We then compare the thermocell device performance.

## Results and Discussion

One of the favorable factors for choosing the iron perchlorate electrolyte was that the high solubility of iron perchlorate salts in water^28^ such that its use can increase the output current of the TEC and at the same time decrease the thermal conductivity of electrolyte. The non-volatile and stable nature of the perchlorate anion is another favorable factor, while highly toxic hydrogen cyanide gas can evolve from the cyanide electrolyte when decomposed thermally or under acidic conditions^29^. Because of these favorable factors, iron perchlorate electrolyte was chosen for the investigation in this work.

**Figure 1:**
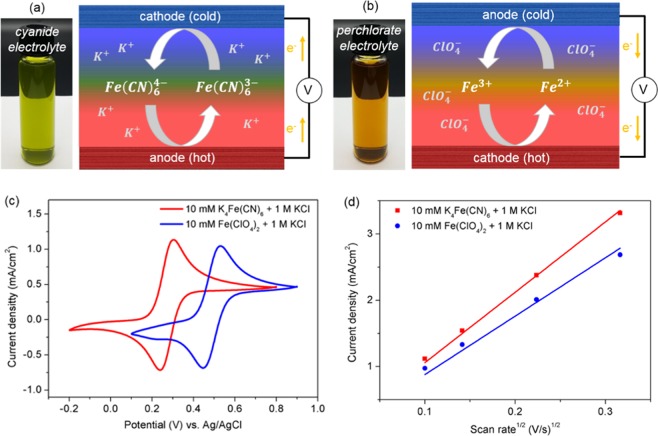
Schematics of redox reactions of (**a**) Fe(CN)_6_^3−^/Fe(CN)_6_^4−^ and (**b**) Fe^2+^/Fe^3+^ redox couples at hot and cold electrodes. (**c**) Cyclic voltammograms of the electrolyte solutions of 10 mM K_4_Fe(CN)_6_ and Fe(ClO_4_)_2_ with 1.0 M KCl as the supporting electrolyte. (**d**) Peak current versus scan rate square-rooted.

Figure 1a,b are the schematics of the redox reactions of Fe(CN)_6_^3−^/Fe(CN)_6_^4−^ and Fe^2+^/Fe^3+^, respectively, driven by a temperature difference applied to the electrodes at both ends of TEC. An optical image of each electrolyte is shown in the right panel in the figure. While the oxidation reaction of Fe(CN)_6_^4−^ to Fe(CN)_6_^3−^ occurs at the hot electrode (*i.e*., anode) and the reduction of Fe(CN)_6_^3−^ to Fe(CN)_6_^4−^ occurs at the cold electrode (*i.e*., cathode), the redox reaction for Fe^2+^/Fe^3+^ redox couple occurs at the opposite electrodes, *i.e*., Fe^2+^ is oxidized to Fe^3+^ at the cold electrode and Fe^3+^ is reduced to Fe^2+^ at the hot electrode. Therefore, the sign of temperature coefficient of redox potential of Fe^2+^/Fe^3+^ redox couple is opposite to Fe(CN)_6_^3−^/Fe(CN)_6_^4−^. This feature should make it possible to connect the TEC with that utilizing Fe(CN)_6_^3−^/Fe(CN)_6_^4−^ redox couple in series path oriented in a flip-flop configuration^18,26,27^.

The redox reactions leading to the generation of electricity have to be reversible for continuous operation of the TEC. Cyclic voltammetry (CV) was used to check the reversibility of electrochemical reactions of the perchlorate electrolyte with platinum as the electrode. The cyclic voltammograms of the electrolyte solutions of 10 mM K_4_Fe(CN)_6_ and Fe(ClO_4_)_2_, obtained with the conventional three electrode configuration, are compared in Fig. 1c with 1.0 M KCl as the supporting electrolyte. The figure shows that the separation between reduction and oxidation peaks (ΔE_p_) of the perchlorate electrolyte solution is 74 mV, which is slightly higher but comparable to the cyanide electrolyte solution (ΔE_p_~62 mV). Figure 1d shows the dependence of the peak current versus the scan rate square-rooted as a function of the scan rate from 10 to 100 mV/sec (see also the cyclic voltammograms of Fig. S1a,b in Supplementary Information). The relationship between the scan rate square-rooted and the pick current density is highly linear for both electrolytes, implying that the redox reactions are limited by free diffusion in the reaction. Therefore, it is concluded from the CV analysis that the redox reaction of the perchlorate electrolyte appears to be quasi-reversible and self-regenerative that the reaction product formed at one electrode becomes a reactant at the other electrode, as with the cyanide electrolyte.

**Figure 2:**
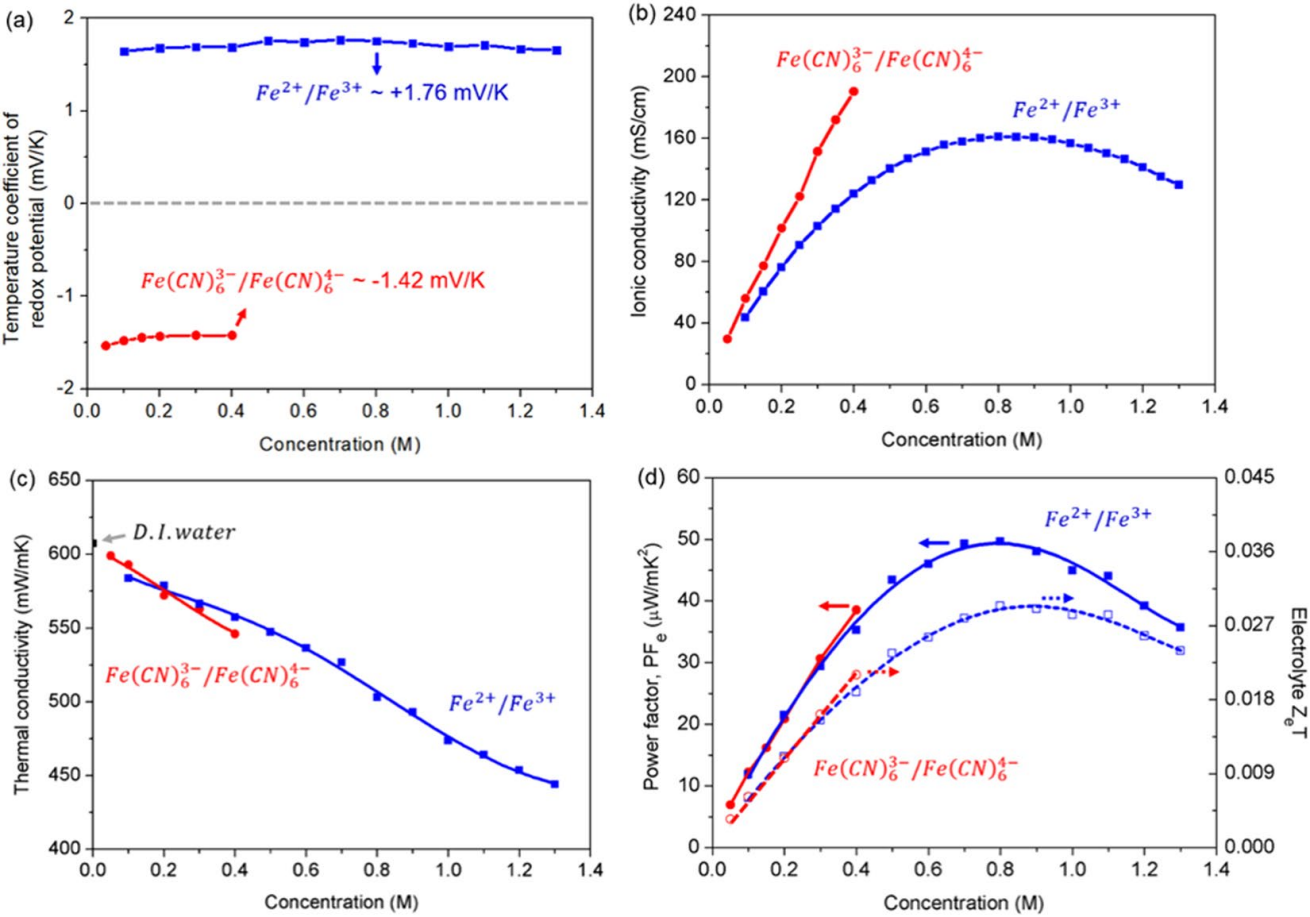
(**a**) Comparison of temperature coefficients of redox potential between Fe(CN)_6_^3−^/Fe(CN)_6_^4−^ and Fe^2+^/Fe^3+^ redox couples in water as a function of their concentrations. Comparison of (**b**) ionic and (**c**) thermal conductivity of the electrolytes. (**d**) Comparison of power factor and figure of merit between the electrolytes.

For a comparison of electrolyte performance between the proposed perchlorate electrolyte and the benchmark cyanide electrolyte, we determined experimentally (see Experimental Section involving non-isothermal electrochemical measurement) the temperature coefficients of Fe(CN)_6_^3−^/Fe(CN)_6_^4−^ and Fe^2+^/Fe^3+^ redox couples as a function of their concentrations. As shown in Fig. 2a, the α of Fe(CN)_6_^3−^/Fe(CN)_6_^4−^ electrolyte was measured to be −1.42 mV/K at a saturated concentration of 0.4 M, which is consistent with previous reports^12,13,24^. A α of +1.76 mV/K, which is 23% higher in absolute magnitude than that of the 0.4 M Fe(CN)_6_^3−^/Fe(CN)_6_^4−^, was measured at 0.8 M concentration of Fe^2+^/Fe^3+^ electrolyte (see also Supporting Information involving voltage changes as a function of temperature). A slightly lower α of +1.66 mV/K was measured near the saturation concentration of 1.3 M Fe^2+^/Fe^3+^.

In fact, the temperature coefficient of perchlorate electrolyte is the highest among the reported Fe^2+^/Fe^3+^ salt systems with different counter ions (*e.g*., α of +0.13 mV/K for ammonium iron sulfate, +0.29 mV/K for iron sulfate, +1.35 mV/K for iron triflate, and +1.34 mV/K for iron nitrate)^30^, as well as higher compared to the cyanide electrolyte. It is clear from the eq. (1) that the sign and magnitude of α are determined by the reaction entropy change (Δs°_rx_) for a given redox reaction^31–33^. The Δs°_rx_ of transition metal redox couples, including Fe^2+^/Fe^3+^, was described by the Born model^34–36^, where each redox active center can be treated as a sphere, having the first solvation layer dielectrically saturated. However, this assumption fails to capture the non-covalent interactions that account for the difference in α values of Fe^2+^/Fe^3+^ redox system with different counter ions. Actually, the non-covalent interactions between redox-counter ions and solvent molecules, and those among solvent molecules affect the solvation structures and correspondingly result in the changes of reaction entropy of redox couples^37^. The Δs°_rx_ was found to increase linearly with greater structural entropy for counter ions^37^. It should be noted in this regard that perchlorate ion is known as a strong water structure breaker^38,39^ in the electrolyte. Therefore, a high Δs°_rx_ in the perchlorate electrolyte could be attributed to the altered solvation shells of Fe^2+^/Fe^3+^ redox species due to the non-covalent interactions, resulting in a high α of the perchlorate electrolyte.

The ionic and thermal conductivities of both electrolytes are compared in Fig. 2b,c, respectively. Figure 2b shows that the ionic conductivity of Fe(CN)_6_^3−^/Fe(CN)_6_^4−^ electrolyte increases monotonically up to the saturation concentration at which the conductivity is 190.3 mS/cm. In contrast, the ionic conductivity goes through a maximum with the concentration in the case of Fe^2+^/Fe^3+^ electrolyte, reaching the highest value of 161 mS/cm at 0.8 M, then decreasing to 129.7 mS/cm at 1.3 M due to strong ionic interactions at high concentration. It seems unlikely that the measured ionic conductivity directly corresponds to the conductivity of TEC device because of irreversible voltage losses occurred during TEC operation. The voltage loss is generated by three primary internal overpotentials, such as activation, ohmic and mass transport overpotentials. Discussion on the conductivity of TEC device is provided in Supplementary Information in conjunction with the internal resistance extracted from the current-voltage curves of TECs.

Figure 2c shows the thermal conductivities of the Fe^2+^/Fe^3+^ and Fe(CN)_6_^3−^/Fe(CN)_6_^4−^ electrolytes that were determined as a function of the concentration. Since the output voltage of TEC is proportional to the temperature difference between the two electrodes, a lower thermal conductivity of electrolyte is highly desirable to lower the heat transport losses through the TEC, thereby enhancing the conversion efficiency of TEC. As shown in the figure, the thermal conductivities of both electrolytes decrease with increasing concentration. Therefore, the lowest conductivity occurs at the saturation concentration, the value being 546 mW/m∙K for the Fe(CN)_6_^3−^/Fe(CN)_6_^4−^ electrolyte at 0.4 M, and 503 and 444 mW/m∙K at the concentrations of 0.8 and 1.3 M, respectively, for the Fe^2+^/Fe^3+^ electrolyte.

Now that the temperature coefficient of redox potential (α), ionic conductivity (σ), and thermal conductivity (λ) have all been determined, we can evaluate the electrolytes in terms of ionic power factor and ionic figure of merit. For the purpose, one can adopt the performance measures of TE materials for the evaluation of TEC electrolytes, particularly in view of the fact that TEC has the same equivalent circuit as TE. Accordingly, we define the ionic power factor (PF_e_) and the ionic figure of merit (Z_e_T) as follows:

2 $${{\rm{PF}}}_{{\rm{e}}}={{\rm{\alpha }}}^{2}\sigma $$

3 $${{\rm{Z}}}_{{\rm{e}}}T=\frac{{{\rm{\alpha }}}^{2}\sigma }{\lambda }T$$

where T is an absolute temperature of electrolyte. A higher PF_e_ would indicate a larger electrical power, but not necessarily a better efficiency for the energy conversion. This energy conversion efficiency of a given electrolyte would be represented by the ionic figure of merit.

Figure 2d shows the calculated values of PF_e_ and Z_e_T of the two electrolytes at room temperature (~25 °C). For the Fe(CN)_6_^3−^/Fe(CN)_6_^4−^ electrolyte, the maximum occurs at 0.4 M for both measures of performance, the maximum being 38.5 μW/m∙K for PF_e_ and 0.021 for Z_e_T. This 0.4 M concentration has mostly been used in TEC studies^12–16,18^. In the case of the proposed Fe^2+^/Fe^3+^ electrolyte, the maximum occurs at 0.8 M, delivering a 28% increase in PF_e_ (49.6 μW/m∙K^2^) and a 40% increase in Z_e_T (0.029) compared to the Fe(CN)_6_^3−^/Fe(CN)_6_^4−^ electrolyte. Therefore, it can be concluded that the optimized 0.8 M Fe^2+^/Fe^3+^ electrolyte would deliver a better TEC performance than the benchmark 0.4 M Fe(CN)_6_^3−^/Fe(CN)_6_^4−^ electrolyte could.

**Figure 3:**
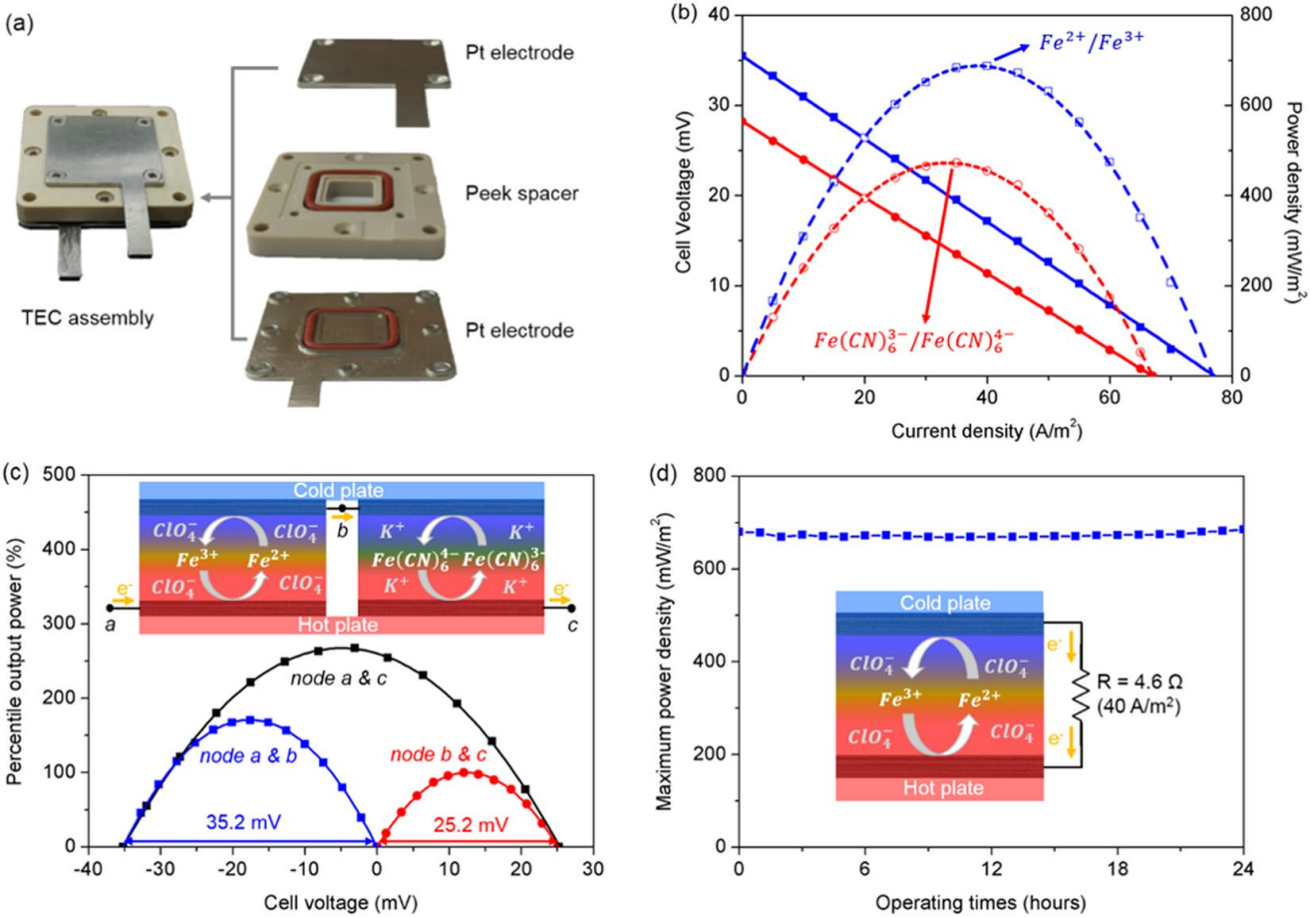
(**a**) TEC configuration used for device performance evaluation. (**b**) Performance of the TEC with 0.8 M Fe^2+^/Fe^3+^ electrolyte against that with 0.4 M Fe(CN)_6_^3−^/Fe(CN)_6_^4−^ electrolyte. (**c**) Power generation of the combined cells of a p-type half-cell using 0.4 M cyanide electrolyte and an n-type half-cell using 0.8 M perchlorate electrolyte that are connected in series. (**d**) Long-term operation stability of the perchlorate electrolyte TEC at an output current density of 40 A/m^2^ for a day.

To validate that the 0.8 M perchlorate electrolyte would be more effective in TEC power generation than the 0.4 M cyanide electrolyte, we fabricated TEC devices using platinum electrodes with an area of 1 cm^2^. The electrode was prepared by depositing 200 nm thick platinum with 5 nm chromium adhesion layer on stainless steel collecting electrodes using thermal evaporation. The inter-electrode spacing was fixed at 5 mm. Figure 3a shows the TEC configuration that was used for the performance evaluation. Since the main objective was to evaluate the electrolyte performance for a given temperature difference rather than to maximize the power density, we applied a small temperature difference of 20 °C to the cells, which was calculated using each open-circuit voltages and the observed temperature coefficients in Fig. 2a. The operating temperature (*i.e*., the average temperature between the two electrodes) of both the cells was set equal to 25 °C, thus the temperature conditions for the reaction rate are the same for each hot or cold electrode in both cells.

Figure 3b shows the power generation capability of the TEC device with 0.8 M Fe^2+^/Fe^3+^ electrolyte (blue curves) against that with 0.4 M Fe(CN)_6_^3−^/Fe(CN)_6_^4−^ electrolyte (red). As expected from the electrolyte performance in Fig. 2d, the Fe^2+^/Fe^3+^ electrolyte is shown to be more effective in TEC power generation than the Fe(CN)_6_^3−^/Fe(CN)_6_^4−^ electrolyte. In fact, the TEC using the Fe^2+^/Fe^3+^ electrolyte yielded a maximum power density (P_max_) of 687.7 mW/m^2^, a 45.4% increase compared to the same device but with Fe(CN)_6_^3−^/Fe(CN)_6_^4−^ electrolyte, for which P_max_ = 472.9 mW/m^2^. In terms of the temperature square normalized specific power density (P_max_/ΔT^2^), the increase is from 1.18 to 1.72 mW/m^2^ K^2^.

To demonstrate voltage and power scaling, two identical TECs but with the perchlorate electrolyte in one TEC, an n-type electrolyte, at the optimized concentration of 0.8 M and in the other the cyanide electrolyte, a p-type, at the optimized concentration of 0.4 M, were connected in series based on the flip-flop configuration^18,26,27^, as shown in the inset of Fig. 3c. The perchlorate and the cyanide cells are electrically connected in series with electrons passing from hot electrode to cold electrode through the n-type perchlorate cell and from cold electrode to hot electrode through the p-type cyanide cell. This interconnection, analogous to commercial p-n thermoelectrics, removes the need for the wiring between hot and cold junctions, thereby eliminating the associated thermal transport path and simplifying fabrication.

Figure 3c shows the percentile output powers normalized with respect to the output power of the cyanide cell. The maximum output power from the combined TECs is achievable when the external load resistance is set equal to the sum of the internal resistance of each cell (corresponding to 4.6 Ω for the perchlorate and 4.2 Ω for the cyanide cell). When the ΔT of 20 °C was applied to the combined TECs and an external load of 8.8 Ω was connected to the circuit, the maximum output power (266.5%) approximately equals the sum of the maximum output powers of the two cells when they are individually operated (100% for the cyanide cell and 170.4% for the perchlorate cell). A slight difference in the maximum powers (~4%) might be caused by the resistance of the wire connecting the TECs.

The V_oc_ from the combined TECs was measured to be 60.4 mV between the nodes *a* and *c*, which is equal to the sum of the V_oc_s of the perchlorate and cyanide cells as shown in the figure. However, it should be noted that, although the V_oc_ for the perchlorate cell was 35.2 mV (between the nodes *a* and *b*), as expected from ΔT of 20 °C and its temperature coefficients, the V_oc_ for the cyanide cell was only 25.2 mV (between the nodes *b* and *c*), corresponding to a ΔT of ~17.7 °C. Accordingly, the effective temperature coefficient for the present cell was determined to be 3.02 mV/K, not 3.18 mV/K which is the result of simply adding the α of each p-type and n-electrolyte. Given the purpose of a series connection to improve the output voltage, the performance of the combined TECs presented here is limited by a higher thermal conductivity of the 0.4 M cyanide electrolyte, compared to the 0.8 M perchlorate electrolyte, as shown in Fig. 2c. More discussion is provided in Supplementary Information.

The stability of the redox reaction of the newly proposed electrolyte is of practical interest for continuous operation of the TEC. A long-term operation stability test was conducted for a day, in which an output current density of 40 A/m^2^ was maintained constant that corresponds to the maximum power density of 687.7 mW/m^2^. As shown in Fig. 3d, the output power from the TEC is maintained nearly constant with a fluctuation of 2.7% in the peak to peak variation of the maximum power density, showing the stability of the electrolyte with on-time.

In summary, we have found the proposed electrolyte of iron perchlorate to be quite effective in converting waste heat to electricity. The ionic power factor and figure of merit for this new electrolyte are higher by 28% and 40%, respectively, than those for the cyanide electrolyte that has been the benchmark for almost half a century. For a basic simple thermocell device that is configured the same but with the different electrolyte for comparison, the electric power generated by the proposed electrolyte is 45% higher than the power delivered by the benchmark electrolyte for a small temperature difference of 20 °C. The perchlorate electrolyte with cationic redox reaction, an n-type electrolyte, compliments the anionic cyanide electrolyte, a p-type. This feature should make it possible to connect the TECs in series in a flip-flop configuration for p-n TECs, as in p-n thermoelectrics. In view of the tremendous progress made in the thermocell performance with the introduction of the cyanide electrolyte, the proposed electrolyte bodes well for the significant advances that could be made with its introduction.

## Methods

### Electrolyte preparation

Various 1:1 concentrations of iron (II) perchlorate (Iron(II) perchlorate hydrate 98%, Fe(ClO_4_)_2_·xH_2_O, Sigma Aldrich) and iron(III) perchlorate (Iron(III) perchlorate hydrate crystalline, Fe(ClO_4_)_3_·xH_2_O, Sigma Aldrich) electrolyte solutions were prepared at concentration of 0.1 to 1.3 M (which is close to saturation) for evaluating electrolyte performance. 1:1 concentration of potassium ferricyanide (K_3_Fe(CN)_6_, Sigma Aldrich) and potassium ferrocyanide (K_4_Fe(CN)_6_·3H_2_O, Sigma Aldrich) electrolyte solutions were prepared at a concentration of 0.05 to a saturated concentration of 0.4 M. Provided concentrations here and elsewhere are total molar concentrations. All solutions were prepared using water from a high purity deionization system (Ultra 370, YOUNG LIN instruments) and were degassed before use by bath sonication. To avoid the effect of electrolyte degradation, the freshly prepared electrolytes were immediately incorporated for all measurements.

### Measurement of temperature coefficient of redox potential

The temperature coefficient of electrode potential of the electrolytes was investigated by measuring the temperature dependence of the potential difference using a non-isothermal cell, as described in our previous work^13,14^. Briefly, a U-shaped cell was used that consists of two half-cells surrounded by water pockets. The temperature of each compartment was controlled by the circulating cold and hot water stored in thermostatic baths (AD-RC08, AND Korea), providing a ±0.1 °C control of the water temperatures. A half-cell has a diameter of 1.0 cm and the distance between half-cells is 8.5 cm long to inhibit the thermal conduction between cell. Platinum wires were used to measure a potential difference generated by the temperature difference of half-cells. A thermocouple (K-type, TM-947SD, LT Lutron) was inserted in close proximity to the electrode for each half-cell. An output voltage from the cell was recorded using a multimeter (Keithley 2000, Tektronix) to measure a temperature coefficient of electrode potential of electrolyte.

### Measurement of thermal conductivity of electrolyte

In measuring the thermal conductivity of electrolytes, strict control over the measurement environment is required to obtain accurate and repeatable results. We prepared 40 mL of electrolyte sample in a glass vial and inserted the end of the sensor needle (KS-1 sensor) into the center of the electrolyte. The sensor needle was oriented vertically during measurement to prevent free convection. Then, we allowed the electrolyte and the needle to equilibrate with the ambient temperature for one hour before the measurement. All measurements were made in an environment controlled at 25 °C. The sensor applied a very small amount of heat to the sample during the measurement to minimize the problem of free convection. The duration of the readout time was set to 1.0 minute to minimize the amount of heat applied to the sample. To eliminate forced convection effects on the results, all measurements were carried out on a vibration isolation table.

### Instrumental

Ionic conductivity and thermal conductivity of electrolytes were measured using a conductivity meter (S-230, Metter Toledo) and a thermal properties analyzer (KD2 Pro, Decagon Devices, Inc.), respectively. All data from CV, EIS, current and voltage (I-V) output from the cell were measured using a computer controlled voltage–current meter (CS310, Corrtest instruments) with 10 μV potential resolution and 10 pA current sensitivity from −10 to 10 V.

## Supplementary information

supporting information
