# Supplementary material for: Iron (II/III) perchlorate electrolytes for electrochemically harvesting low-grade thermal energy
Source: Sci Rep. 2019 Jun 18;9:8706. doi: 10.1038/s41598-019-45127-w (PMC6582052; doi:10.1038/s41598-019-45127-w)
Supplement: Supplementary file 1 — supporting information [file 41598_2019_45127_MOESM1_ESM.docx]

Supporting Information

Iron (II/III) perchlorate electrolytes for electrochemically harvesting low-grade thermal energy

Ju Hyeon Kim^1^, Ju Hwan Lee^1^, Ramasubba Reddy Palem^1^, Min-Soo Suh^2^, Hong H. Lee^3^ & Tae June Kang^1,*^

^1^ Department of Mechanical Engineering, INHA University, Incheon 22212, South Korea

^2^ Energy Efficiency and Materials Research Division, Korea Institute of Energy Research, Daejeon 34129, South Korea

^3^ School of Chemical and Biological Engineering, Seoul National University, Seoul 151-744, South Korea

* tjkang@inha.ac.kr

**Recent advances in thermocell performance**

A TEC is a non-isothermal electrochemical cell which has a simple structure of an electrolyte sandwiched between two non-active electrodes. Studies on TECs had mainly centered around the electrolyte of ferric/ferrous cyanide (Fe(CN)_6_^3-^/Fe(CN)_6_^4-^) with platinum electrodes. With the efforts on developing inexpensive but highly efficient electrodes and high-performance electrolytes, remarkable advances have been made in the TEC performance, which render TEC technologies commercially attractive, considering that the Carnot relative efficiency for commercial viability is 2 to 5%^1^.

For TEC electrodes, the TEC with platinum electrodes reached only a conversion efficiency relative to the Carnot limit (η_rel_) of 0.6% and a maximum power density (P_max_) of 3.6 mW/m^2^ at an inter-electrode temperature difference (ΔT) of 20^o^C^2^. Remarkable advances have been made in the TEC performance with the introduction of highly porous, high surface area and conductive electrodes. By incorporating carbon nanotube (CNT) as an electrode material, the TEC performance was increased to η_rel_=1.4% and P_max_=1.8 W/m^2^ at a ΔT of 60^o^C^3^. Recently, the performance was further improved using a highly aligned CNT aerogel sheet, reaching η_rel_=3.95% and P_max_=6.6 W/m^2^ at a temperature difference of 51^o^C^4^.

For TEC electrolytes, theoretical and experimental studies proceeded to understand a temperature dependency of the electrode potential for a redox reaction (*i.e.*, the ionic Seebeck effect)^5-7^. It was revealed from the studies that the ionic Seebeck effect depends mainly on the size and charge type of the redox complex ions once the specific nature of the solvent is included^5,6^. An aqueous electrolyte with Fe(CN)_6_^3-^/Fe(CN)_6_^4-^ redox couple was first introduced in 1976 and has been the benchmark solution for the TEC electrolyte because of a high ionic Seebeck coefficient of -1.42 mV/K at 0.4 M concentration (which is close to the saturation concentration) and a large exchange current density associated with various electrode materials. Much improvement in the performance has also been realized by introducing solvents or changing the cation in the cyanide electrolyte. The ionic Seebeck coefficient of the cyanide electrolyte was improved to -2.9 mV/K by adding solvents to the electrolyte^7^. Replacement of potassium ferrous cyanide with ammonium ferrous cyanide ((NH_4_)_4_Fe(CN)_6_) in the cyanide redox system increased the saturated concentration of the electrolyte to 0.9 M, which led to a remarkable improvement in a power density up to 12.0 W/m^2^ from the TEC involving an optimized thermal separator^8^. Recently, the significant role of the anion in the iron (II/III) based redox systems has also been demonstrated in terms of their thermogalvanic ability to convert a temperature gradient into electrical power^9^.

**Cyclic voltammetry analysis of the cyanide and perchlorate electrolytes**

Cyclic voltammetry (CV) analysis was conducted to investigate the reversibility of electrochemical reactions of the perchlorate electrolyte. The cyclic voltammograms of the electrolyte solutions of 10 mM K_4_Fe(CN)_6_ and Fe(ClO_4_)_2_, obtained with the conventional three electrode configuration, are compared in Fig. S1a and b with 1.0 M KCl as the supporting electrolyte.


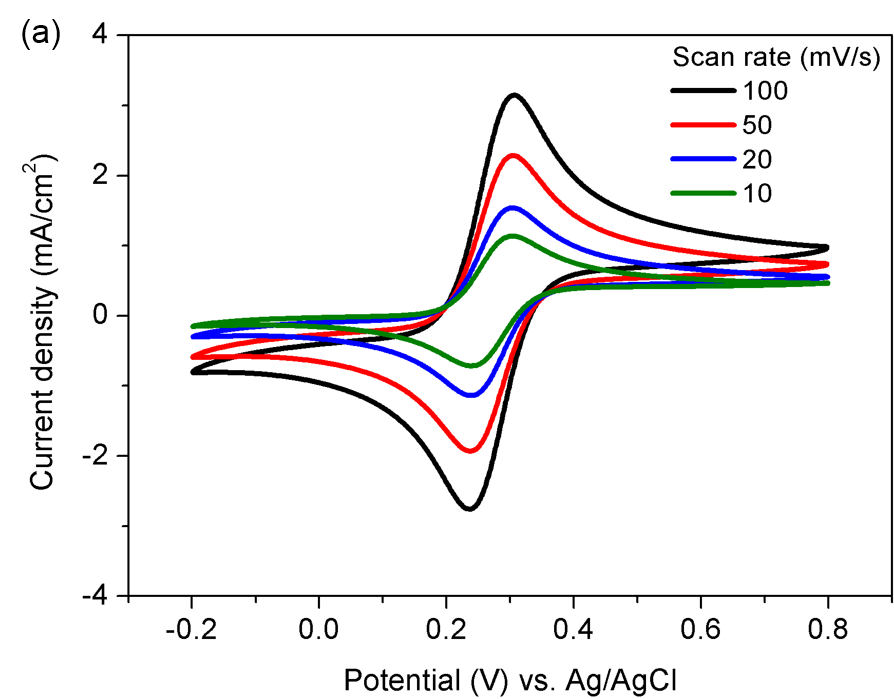


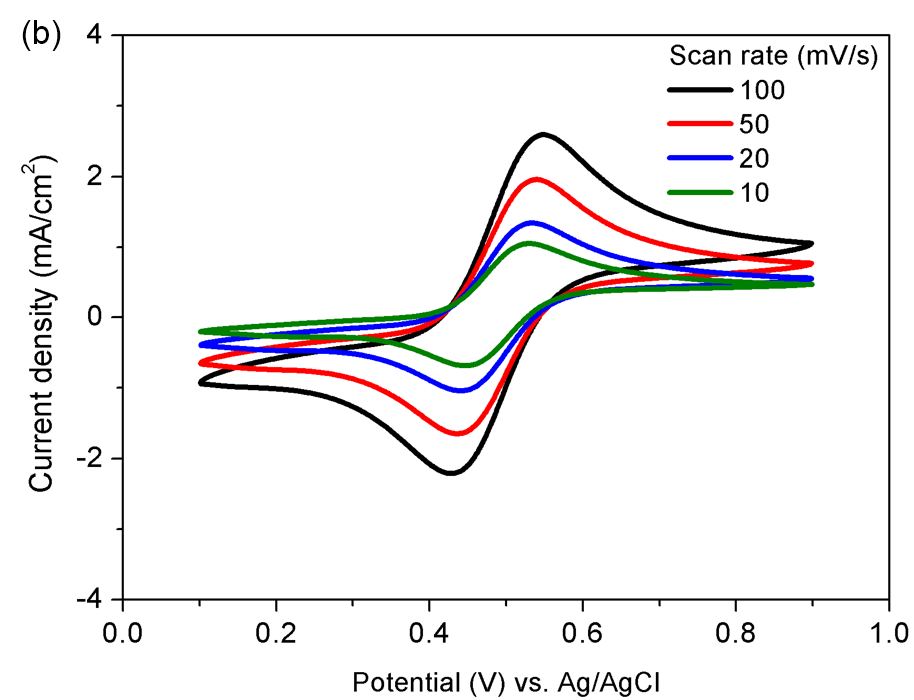


**Figure S1.** Cyclic voltammograms for (a) the K_4_Fe(CN)_6_ and (b) the Fe(ClO_4_)_2_ electrolyte solutions as a function of a scan rate ranging from 10 to 100 mV/sec

**Voltage changes as a function of temperature**

The temperature coefficient of redox potential of 0.8 M aqueous electrolyte of iron (II/III) perchlorate was measured using a U-shaped cell that consists of two half-cells surrounded by liquid pockets. The experimental setup for this measurement is provided in Methods involving Measurement of temperature coefficient of redox potential. To evaluate the temperature coefficient, the open-circuit voltage from the cell was recorded using a multimeter by varying the temperature difference between the half-cells. The temperature of the cold side was fixed as being 25°C and the hot side was heated up to 55°C with a temperature change of 5°C.


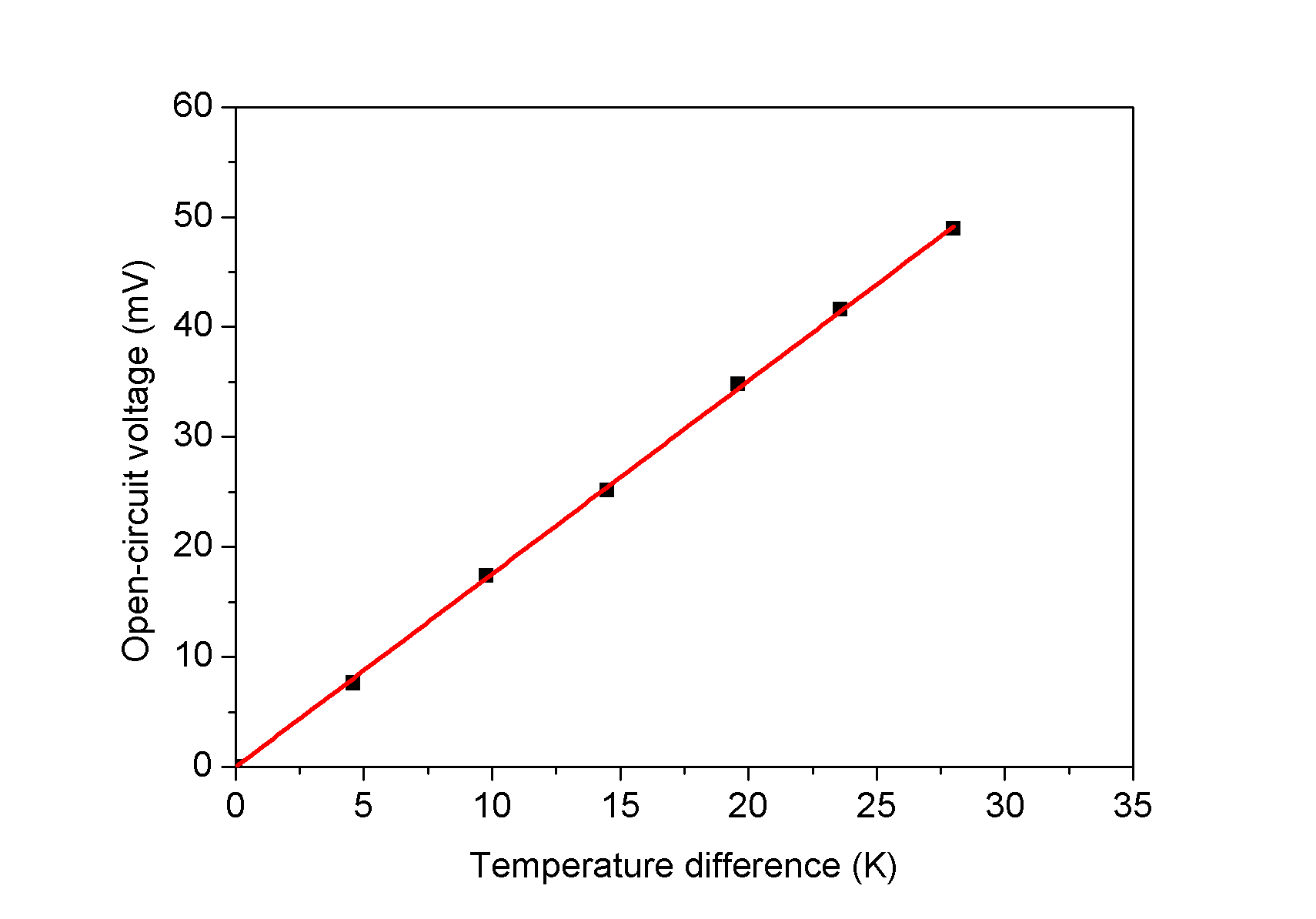


Figure S2. Measurement of the temperature coefficient of redox potential of 0.8 M iron (II/III) perchlorate electrolyte

**Discussion on the conductivity of TEC device**

The conductivity of TEC device can be calculated using an internal resistance (*i.e.*, the slope of a current-voltage (I-V) curve of the TEC) and the geometry of the TEC device. From the I-V curves of Fig. 3b, the internal resistance was measured as 4.6 and 4.2 Ω for the TECs using 0.8 M Fe^2+^/Fe^3+^ and 0.4 M Fe(CN)_6_^3-^/Fe(CN)_6_^4-^ electrolytes, respectively, which correspond to the TEC device conductivity of 108.7 and 119 mS/cm. It should be noted that the device conductivity is much lower than the ionic conductivity (161 mS/cm for 0.8 M Fe^2+^/Fe^3+^ and 190.3 mS/cm for 0.4 M Fe(CN)_6_^3-^/Fe(CN)_6_^4-^) that was measured using an ion conductivity meter in Fig. 2b.

This difference between the TEC conductivity and the ionic conductivity of electrolyte arises from irreversible voltage losses occur during TEC operation. Voltage losses from the equilibrium voltage or the open-circuit voltage are generated by three primary internal overpotentials, such as activation (voltage loss to overcome the activation barrier associated with the reactions at the electrode), ohmic (resistive loss in the electrolyte and in the electrode) and concentration (mass transport loss arising due to the concentration gradient in the electrolyte as well as in porous electrodes) overpotentials. The internal resistance of the TEC device is comprehensively determined by these overpotentials. Therefore, the measured ionic conductivity does not directly correspond to the conductivity of TEC device.

Since the performance of TECs is greatly affected by the electrode, which is associated with the overpotential mentioned above, it is necessary to distinguish the performance metrics of the electrolyte from those of the device. For the purpose, we adopt the performance measures of TE materials, particularly in view of the fact that TEC has the same equivalent circuit as TE. Accordingly, we define the ionic power factor and the ionic figure of merit for the evaluation of TEC electrolytes.

**Discussion on the performance of the combined TECs**

Since the same current must flow through both the n-type and the p-type cells in series-connected TECs, the output power from the combined TECs might be limited by the current mismatch of each cell that produces the maximum power, which is expressed as the mismatch between the V_oc_ ratio (V_oc_ from the perchlorate cell/V_oc_ from the cyanide cell ~ 1.4) and the internal resistance ratio (R_int_ of the perchlorate cell/R_int_ of the cyanide cell ~ 1.15)). However, the maximum power loss for the current mismatch presented here is negligible (a 0.9% decreas in the maximum power). Therefore, the difference in the maximum powers might be caused by the resistance of the wire connecting the TECs.

The ionic Seebeck coefficient of the combined TEC is not the same as the result of simply adding the ionic Seebeck coefficient of each p-type and n-electrolyte. It can be ascribed to a higher thermal conductivity of the 0.4 M cyanide electrolyte, compared to the 0.8 M perchlorate electrolyte. It implies that an effort should be made to match the thermal conductivity of the electrolytes used in the TEC series connection, as well as to match the output current of each cell that can deliver the maximum output power. If the purpose is only to increase the output voltage through thermal conductivity matching, it would be possible to use a 0.5 M perchlorate electrolyte showing a similar thermal conductivity with the 0.4 M cyanide electrolyte, as shown in Fig. 2c. However, this should not be an effective way in terms of power generation.

Efforts to explore new high-performance p-type electrolytes are required to take advantage of the maximum performance of the perchlorate electrolyte. For example, ammonium ferrous cyanide ((NH_4_)_4_Fe(CN)_6_)/ potassium ferric cyanide redox system shows a high saturation concentration of 0.9 M in water, and thus, can provide a lower thermal conductivity than the 0.4 M cyanide electrolyte. This electrolyte can be more complementary in serial interconnection with the 0.8 M perchlorate electrolyte. Another way to match the thermal conductivity of the electrolyte is to introduce a thermal separator into the TEC. As reported in our previous study^7^, thermal separators with appropriate porosity and density can lower the effective thermal conductivity of the electrolyte, and thus, increase the temperature gradient inside the cell with minimal impact on the ionic conductivity of the electrolyte.

**References**

1 Quickenden, T. & Mua, Y. A review of power generation in aqueous thermogalvanic cells. *Journal of The Electrochemical Society* **142**, 3985-3994 (1995).

2 Mua, Y. & Quickenden, T. Power conversion efficiency, electrode separation, and overpotential in the ferricyanide/ferrocyanide thermogalvanic cell. *Journal of The Electrochemical Society* **143**, 2558-2564 (1996).

3 Hu, R. *et al.* Harvesting waste thermal energy using a carbon-nanotube-based thermo-electrochemical cell. *Nano letters* **10**, 838-846 (2010).

4 Im, H. *et al.* High-efficiency electrochemical thermal energy harvester using carbon nanotube aerogel sheet electrodes. *Nature communications* **7**, 10600 (2016).

5 Yamato, Y., Katayama, Y. & Miura, T. Effects of the interaction between ionic liquids and redox couples on their reaction entropies. *Journal of The Electrochemical Society* **160**, H309-H314 (2013).

6 Hupp, J. T. & Weaver, M. J. Solvent, ligand, and ionic charge effects on reaction entropies for simple transition-metal redox couples. *Inorganic Chemistry* **23**, 3639-3644 (1984).

7 Bratsch, S. G. Standard electrode potentials and temperature coefficients in water at 298.15 K. *Journal of Physical and Chemical Reference Data* **18**, 1-21 (1989).

8 Zhang, L. *et al.* High Power Density Electrochemical Thermocells for Inexpensively Harvesting Low‐Grade Thermal Energy. *Advanced Materials* **29**, 1605652 (2017).

9 Buckingham, M. A., Marken, F. & Aldous, L. The thermoelectrochemistry of the aqueous iron (ii)/iron (iii) redox couple: significance of the anion and pH in thermogalvanic thermal-to-electrical energy conversion. *Sustainable Energy & Fuels* **2**, 2717-2726 (2018).
